# Successful permanent pacemaker explantation after diagnosis and treatment of Lyme carditis complete heart block: a case report

**DOI:** 10.1093/ehjcr/ytad576

**Published:** 2023-11-24

**Authors:** Christopher R Messner, Richard S Amara

**Affiliations:** Department of Medicine, University of Maryland, Baltimore, MD, USA; Department of Cardiology, University of Maryland, Baltimore, MD, USA

**Keywords:** Arrhythmias, Pacemaker, Complete heart block, Lyme carditis, Case report

## Abstract

**Background:**

Lyme carditis (LC) complete heart block (CHB) is typically treated with i.v. antibiotics without requiring permanent pacing. In patients with high degree atrioventricular (AV) block, suspicious index in Lyme carditis (SILC) scoring is highly sensitive for diagnosing LC.

**Case summary:**

We present a case of CHB where a permanent pacemaker (PPM) was implanted prior to LC diagnosis. Suspicious index in Lyme carditis score was 2 at the time of exam, indicating a low risk for LC. However, per further discussion at follow-up, his score was retroactively increased to an intermediate risk of 4 and Lyme titres returned positive. An outpatient oral antibiotic regimen was given, and 2 months later, the patient had <0.1% V-pacing on interrogation with a subsequent unremarkable event monitor. The pacemaker was removed after considerations ensuring full conduction recovery. The patient is doing well at follow-up > 1 year.

**Discussion:**

Lyme carditis spontaneous resolution of CHB is common. Once safe extraction parameters have been established, it is appropriate to engage patients without ongoing pacer requirements about explantation of their PPM. For CHB patients without clear aetiology, SILC scoring may be a predictive measure to help prevent unnecessary PPM placement in the future.

**Learning points**

- Lyme carditis complete heart block (LC CHB) can be treated with an outpatient oral antibiotic regimen resulting in resolution of heart block.
- Suggested safe permanent pacemaker explant parameters including lack of pacing on monitor/interrogation and chronotropic response to stress testing.
- Suspicious index in Lyme carditis scoring ranks likelihood of LC, and in the future may have predictive value for LC CHB outcomes and need for interventions.

## Introduction

Lyme disease is a tick-borne illness most commonly caused by the bacterium *Borrelia burgdorferi*^1,2^ carried by the Ixodes deer tick endemic to Northeast, North Central, and Mid-Atlantic regions of the USA, as well as Mexico, Europe, and Asia.^1,2^ Presenting signs occur within 1 month of infection and include constitutional symptoms, arthralgias, and an erythema migrans (EM) bulls-eye rash.^2^ Clinical diagnosis can be made if the characteristic EM is present, however diagnostic testing includes antibody testing on an acute-phase serum sample and follow-up convalescent-phase sample if the initial result is negative.^1^

Lyme carditis (LC) is inflammation or direct infiltration of cardiac tissues^3–5^ that results in cardiac symptomatology including heart failure, arrhythmias, and/or myocarditis/pericarditis.^1,2^ Temporary pacemaker insertion is required in around one-third of LC cases presenting with atrioventricular (AV) nodal block, with resolution within ∼10 days of appropriate antibiotics.^3–5^

## Case summary

This patient was a 67-year-old male with medical history including myocardial infarction 25 years ago (unclear territory, no revascularization, no resultant cardiomyopathy) and right bundle branch block (RBBB). The patient presented with chief complaint of 2 days of light-headedness and a syncopal episode with prodrome of facial flushing, headache, nausea, and chest tightness. There were no notable findings or EM on physical exam. Home medications included metoprolol XR 25 mg, atorvastatin 40 mg, and Eliquis 5 mg. Lab work included three troponin I < 0.02 ng/mL (accepted normal < 0.04), BNP 703 pg/mL (accepted normal < 100), and 14 000/mm^3^ leucocytes (accepted normal < 11 000) that resolved the following day.

**Figure 1:**
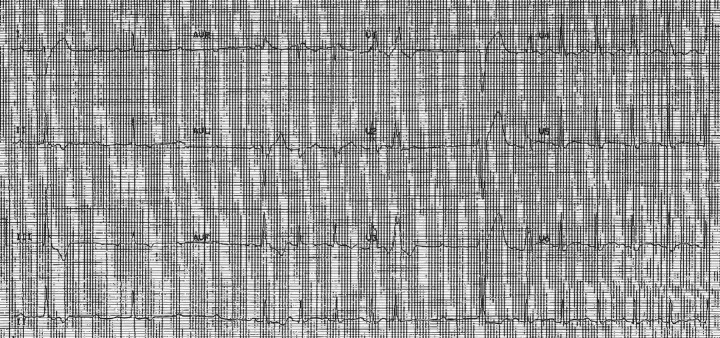
Presenting EKG. Complete heart block observed throughout warranting evaluation for implantation of permanent pacemaker.

**Figure 2:**
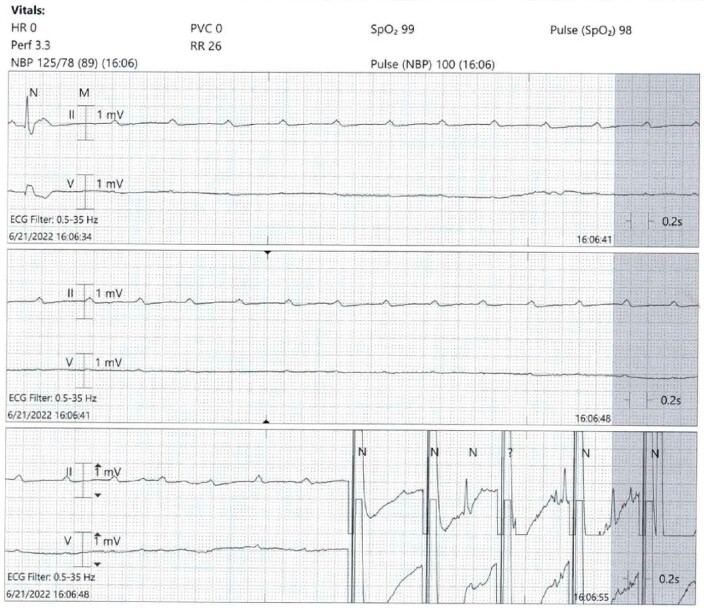
Presenting telemetry strip. Complete heart block observed on telemetry strip in addition to EKG.

Presenting electrocardiogram (EKG) showed sinus rhythm with RBBB and periods of high-grade heart block with ventricular escape complexes (*Figures 1* and *2*). Echocardiogram demonstrated ejection fraction 60–65% without significant abnormality (Supplementary data online, *Video S1*). Subsequently, he developed persistent complete heart block (CHB). Suspicious index in Lyme carditis (SILC) score at time of evaluation was 2 (male, endemic region), low risk.

While Lyme titres were pending, a Medtronic dual-chamber pacemaker was implanted. The patient quickly recovered and was discharged from the hospital. Day 2 after discharge, Lyme titres were positive for acute Lyme on both IgM/G western blot (WB; Spec. > 85%, Sens. 62.3%) and enzyme-linked immunoassay (ELISA; Spec. > 85%, Sens. 53.9%) (Spec. 96.1%, Sens. 59.5%).^6^

On further discussion, the patient admitted to constitutional symptoms 3 weeks prior (fever, fatigue, chills, myalgias), increasing his SILC score to 4, intermediate risk.^7^ He received a 21-day course of oral doxycycline 100 mg twice daily.

## Follow-up and outcomes

At 2 months follow-up, the patient was observed to have 1:1 conduction and had not utilized his pacemaker since 5 days post-implant (interrogation observed V-paced burden in this timeframe of <0.1%, the lowest reportable amount on this generator). Given this, several tests were conducted to assess appropriateness of pacemaker removal. To ensure the pacing burden was truly zero, the pacer was set to ventricular demand pacing at 30 b.p.m. (VVI 30). A subsequent 14-day event monitor showed no heart block episodes or periods of pacing, and during exercise, heart rate increased to >150 b.p.m. with 1:1 conduction. Interrogation continued to show <0.1% ventricular pacing. His pacemaker was successfully explanted 23 weeks after implantation. The patient has done well on >1 year follow-up.

**Figure 3:**
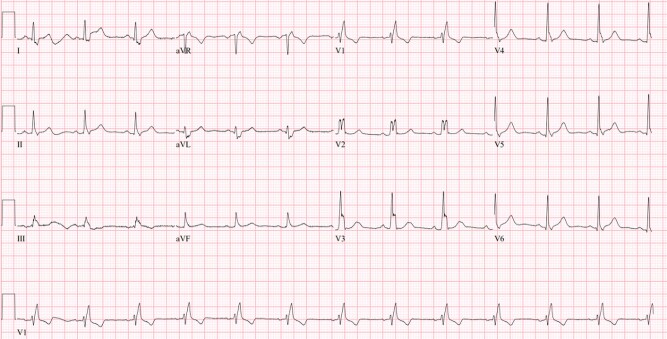
EKG prior to explantation. Conduction abnormality resolved after administration of outpatient antibiotic regimen.

EKG on the day of pacemaker explantation showed normal sinus rhythm with RBBB (*Figure 3*).

## Discussion

The SILC score is derived from a systematic review that demonstrated 100% sensitivity for diagnosis of LC (no control group for specificity) in patients presenting with high degree AV block without clear aetiology.^7^ Constitutional symptoms are worth 2 points, outdoor activity/endemic area 1, male sex 1, tick bite 3, age < 50 1, and EM 4, with rating of 0–2 being low risk, 3–6 intermediate, and 7–12 high. The patient had intermediate SILC of 4 without any other known heart block aetiology. His pre-existing RBBB alone would not clearly explain the development of CHB. Potential causes of CHB include ischaemia, structural defects, electrolyte abnormalities, autoimmune disorders, procedures, and antiarrhythmics, none of which were observed.^8^ In a recent retroactive study, when conduction disease patients were diagnosed with LC via SILC score, 55% of LC patients who received permanent pacemaker (PPM) had resolution of their heart block.^9^ Further research will be required as to how to interpret these findings, including whether low or intermediate risk SILC scores are more indicative of possible CHB resolution with antibiotics. Suspicious index in Lyme carditis score usage could help minimize unnecessary pacemaker implants, or guide decision making on the feasibility of pacemaker explant in this population.

Current recommendations for treatment of LC as per the International Diseases Society of America (IDSA) is i.v. 2 g ceftriaxone for a 10–14-day course followed by possible pacemaker if 1:1 conduction is not restored.^1^ If conduction is restored, then a treadmill stress test can be performed—if the heart rate can reach >120 b.p.m. with 1:1 conduction, initiate oral antibiotics (doxycycline, amoxicillin, azithromycin, or cefuroxime) for a total 14–21-day course with EKG follow-up in 4–6 weeks. Stress testing demonstrating AV Wenckebach < 90 b.p.m. likely requires pacemaker. AV Wenckebach between 90 and 120 b.p.m. warrants repeat stress testing in 4–6 weeks.^1,3^ Our patient received 21 days of oral doxycycline (no i.v. antibiotics), with demonstration of 1:1 conduction at >150 b.p.m. during exercise suggesting full conduction recovery per these guidelines.

Pacemaker insertion comes with complication risk and increased cost burden, however devices removed within the first year of implantation avoid chronic fibrotic changes within the veins and endocardial structures, minimizing long-term complications.^10^ Shared decision making is integral in decision to explant, especially with longer time-courses. Along with the patient, we felt comfortable with pacemaker extraction after antibiotics and the aforementioned rigorous testing. A potential algorithm to define safety in this scenario might include: 0% (or device-reported < 0.1%) V-pacing on a demand V-pacing setting at relatively low rate (VVI 40 or less) on follow-up, a 14 to 30-day event monitor demonstrating no high-grade AV block and confirming no ventricular pacing during this timeframe, and the ability to elevate the heart rate to >120 b.p.m. with 1:1 conduction with exercise. Follow-up could then be conducted after an additional 3 months, then at 6-month intervals.

Current literature actively commenting on oral antibiotics for treatment of LC is sparse. Recently, a study commented on a 59-year-old male patient presenting with CHB who received a PPM, SILC = 4, who was also treated with doxycycline 100 mg orally twice daily for 3 weeks and found to have resolution of heart block, with pacemaker explant at 42 weeks.^11^

## Conclusion

This case report highlights the feasibility of pacemaker explant in LC CHB treated with outpatient oral antibiotics after pacemaker reprogramming and extended event monitoring suggested no continued pacemaker need. While the SILC score is a sensitive diagnostic tool for LC, its functionality as a predictor of resolution of heart block and thus a potential guide to pacemaker explant in this unique population remains an intriguing potential application that deserves further exploration.

## Supplementary Material

ytad576_Supplementary_Data

## Data Availability

The data supporting this article are available throughout the article itself and within the Supplementary material.

## Lead author biography

Christopher Messner is a Penn State College of Medicine graduate now working as an Internal Medicine resident at the University of Maryland Medical Center. He was born and raised near Hershey, PA. Despite the town’s reputation as a purveyor of chocolate, Chris was advised not to partake as he was born with Wolff–Parkinson–White syndrome. Now capable of caffeine consumption after two ablations, he has clinical interests in electrophysiology and arrhythmias secondary to his time as a patient.

## Supplementary material

**Consent:** The author/s confirm that written informed consent for submission and publication of this case report including image(s) and associated text has been obtained from the patient in line with COPE guidance.

**Funding:** None.
